# Integrating a chromatin remodeling layer into single-cell gene regulatory networks

**DOI:** 10.1093/bib/bbaf631.021

**Published:** 2025-12-12

**Authors:** Junsoo Song, Kenji Mizuguchi

**Affiliations:** The University of Osaka; Institute for Protein Research, The University of Osaka

## Abstract

**Aim:**

Gene regulatory networks (GRNs) are essential for uncovering the mechanisms of complex biological systems and diseases. Conventional single-cell GRN methods, however, largely focus on transcription factors (TFs) and their correlation with gene expression. This focus overlooks the role of chromatin accessibility, which arises from the combinatorial activity of pioneer TFs and chromatin-opening cofactors.

**Methods:**

We introduce a systems framework that incorporates an explicit chromatin-remodeling layer upstream of enhancer activation to achieve causal interpretability. First, stable and non-linear cofactor modules are identified, yielding region–module responsibilities that capture combinatorial activity. Second, cell-level priors for each module are estimated from standardized RNA expression of their constituent factors. Third, Bayesian inference predicts single-cell remodeling activity, incorporating enhancer–enhancer dependencies into the likelihood. Finally, posterior module activity is integrated with GRN models to quantify module-driven transcription, producing regulatory paths: cofactor module → enhancer → transcription factor → gene.

**Results:**

We applied this framework to public single-cell multiome datasets of normal and cancerous cells. This application has recovered well-established regulatory interactions and revealed cancer-associated remodeling pathways that may not be evident from motif-based analyses.

**Conclusion:**

Ultimately, the approach is anticipated to contribute to discovering novel regulatory targets within a coherent systems biology paradigm.

